# Supplementary material for: Steroid receptor coactivator-1 modulates the function of Pomc neurons and energy homeostasis
Source: Nat Commun. 2019 Apr 12;10:1718. doi: 10.1038/s41467-019-08737-6 (PMC6461669; doi:10.1038/s41467-019-08737-6)
Supplement: Supplementary file 1 — Supplementary Information [file 41467_2019_8737_MOESM1_ESM.pdf]

Steroid Receptor Coactivator-1 Modulates the Function of Pomc Neurons and Energy Homeostasis

Yang et al.

Supplementary Figure 1. SRC-1 effects in Pomc neurons.

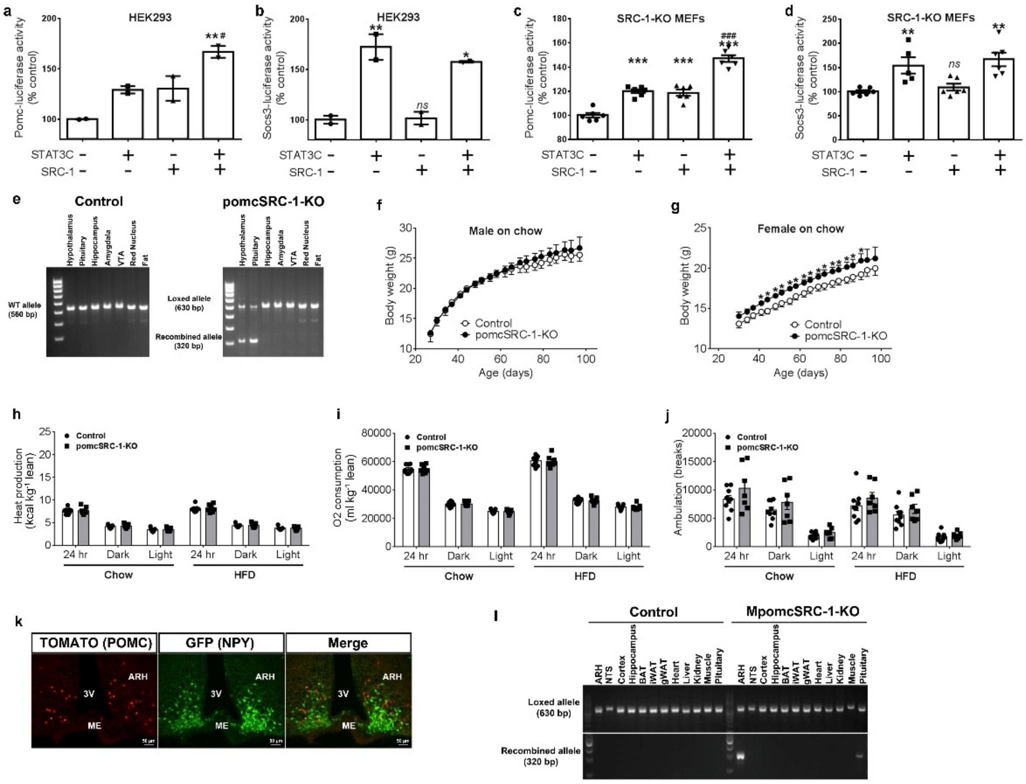

Numbers of mice/experiments are indicated; data are presented as mean ± SEM and compared using T-tests or two-way ANOVA followed by post hoc Sidak tests (#). (A-D) Effects of overexpressed constitutively active STAT3 and SRC-1 on Pomc- or Socs3-luciferase activity in HEK293 cells (a-b) and SRC-1-KO MEF cells (c-d) (n=2-6); * P<0.05, ** P<0.01 and *** P<0.001 vs. empty vectors; # P<0.05 and ### P<0.001 vs. STAT3 alone. (e) Validation of pomcSRC-1-KO mice. PCR amplification of genomic DNA from various brain regions, pituitary and fat. The WT SRC-1 allele (560 bp) was detected in all tissues from a control mouse. The loxed SRC-1 allele (630 bp) was detected in all tissues from a pomcSRC-1-KO mouse, but the recombined SRC-1 allele (320 bp) was only detected in POMC cell-containing tissues (the hypothalamus and pituitary). VTA, ventral tegmental area. (f-g) Body weight of male (f) or female (g) control and pomcSRC-1-KO mice fed regular chow (n=6-13). (h-j) Metabolic phenotypes in male pomcSRC-1-KO mice. 12-week old male control and pomcSRC-1-KO mice with matched body weight, lean mass and fat mass were adapted to the CLAMS chambers. Mice were subjected to a 2-day-chow-2-day-HFD protocol, and chow was replaced by HFD before the onset of dark cycle on day 3. Heat production (h), O2 consumption (i) and ambulatory movement (j) were continuously monitored and averaged for 2-day chow feeding period and for 2-day HFD feeding period (n=7/9). (k) Distribution of TOMATO (induced by *Pomc-CreER*) and GFP (driven by *Npy* promoter) in the ARH of *Pomc-CreER/Rosa26-tdTOMATO/Npy-GFP* mice. No neurons were double labelled, indicating that the mature Pomc neurons targeted by *Pomc-CreER* were segregated from mature Npy neurons. Scale bar=50 µm. 3V, 3rd ventricle; ARH, arcuate nucleus of the hypothalamus; ME, median eminence. (l) PCR amplification of loxed allele and recombined allele in various tissues collected from control vs. Mpomc-SRC-1-KO mice. **Source data are provided as Supplementary Data 1.xlsx.**

Supplementary Figure 2. Leptin-induced effects.

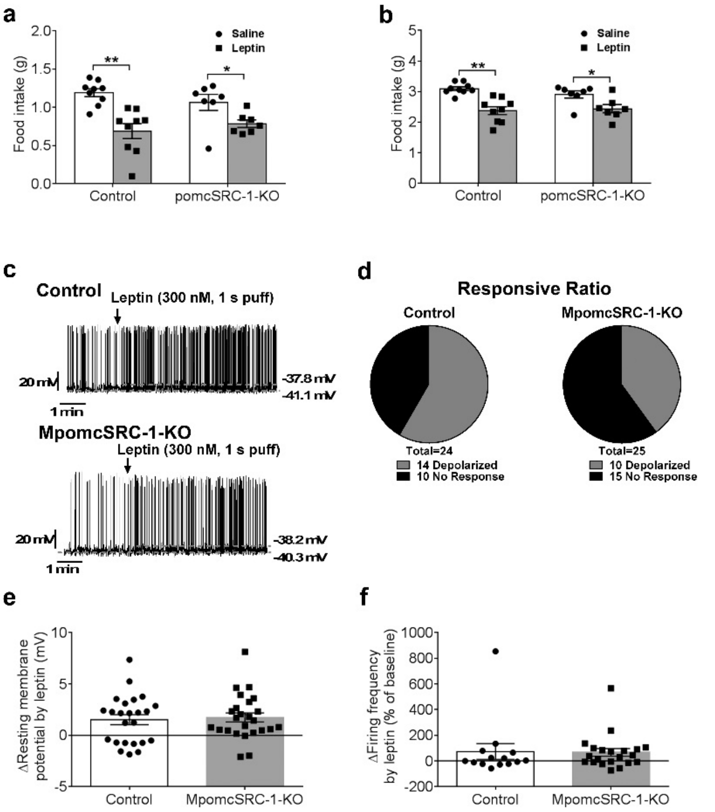

Numbers of mice/neurons are indicated; data are presented as mean ± SEM and compared using T-tests or two-way ANOVA followed by post hoc Sidak tests (#). (a-b) Two-hour fasted mice (12 weeks of age) received i.p. injections of saline/leptin (5 mg/kg) 15 min prior to refeeding and food intake was record for 4 hours (a) or 24 hours (b) afterwards (n=7/9); * P<0.05 and ** P<0.01. (c) Representative traces for leptin-induced depolarization, in the absence of synaptic blockers, in mature Pomc neurons from control mice vs. from MpomcSRC-1-KO mice after one-week HFD feeding. (d) Responsive ratio (depolarization is defined as >2 mV elevations in resting membrane potential, P=0.1994 in $\chi 2$ test). (e-f) Summary quantification of leptin-induced depolarization (e) and increases in firing frequency (f) in two groups (n=14-25). **Source data are provided as Supplementary Data 2.xlsx.**

Supplementary Figure 3. Mutations in SRC-1 impair SRC-1-pSTAT3 interaction.

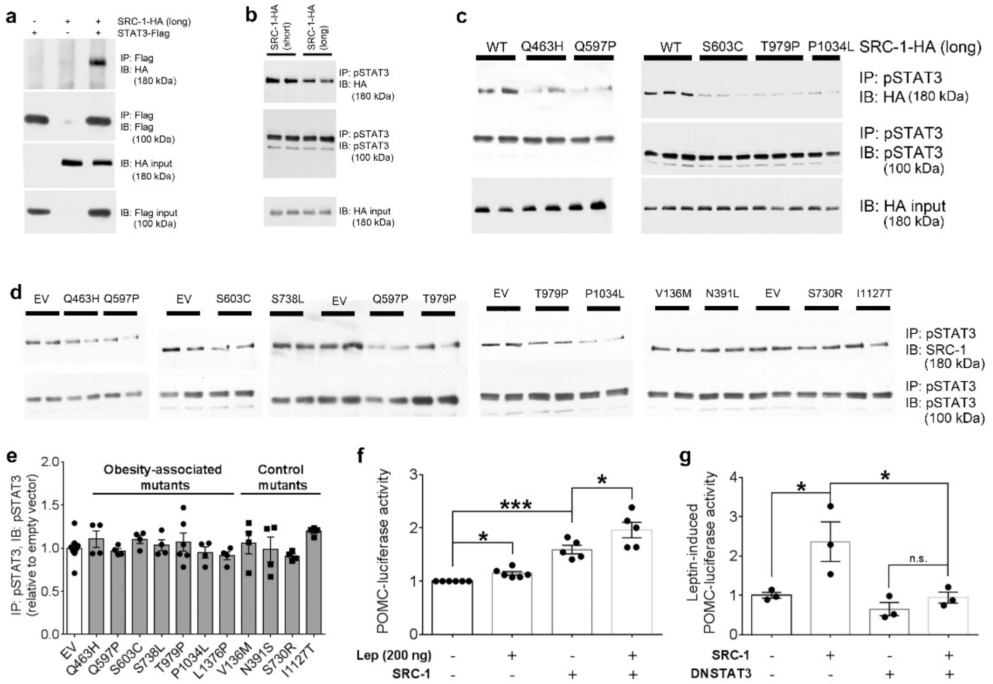

(a) HEK293 cells were transfected with STAT3-Flag alone, WT SRC-1-HA (the long isoform) alone, or the combination of both. Interactions between the STAT3 and SRC-1 were determined by CoIP experiments using anti-Flag and anti-HA antibodies. Representative blots showing interactions between STAT3 and SRC-1, and inputs of HA and Flag. (b) HEK293 cells were transfected with leptin receptor vector and STAT3 vector. Cells were treated with leptin (200 ng/ml, 15 min) to induce phosphorylation of STAT3. pSTAT3 was purified and incubated with long or short isoform of WT SRC-1, and interactions between the pSTAT3 and SRC-1 were determined by CoIP experiments using anti-pSTAT3 and anti-HA antibodies. Representative blots showing interactions between pSTAT3 and SRC-1 and inputs of SRC-1-HA (n=4). (c) HEK293 cells were co-transfected with leptin receptor vector and human STAT3 vector. Cells were treated with leptin (200 ng/ml, 15 min) to induce phosphorylation of STAT3. pSTAT3 was purified and incubated with the long isoform of human SRC-1-HA (WT or obesity-associated mutants), and interactions between the pSTAT3 and SRC-1 were determined by CoIP experiments using anti-pSTAT3 and anti-HA antibodies. (d) HEK293 cells were cotransfected with leptin receptor vector, STAT3 vector, and mutant SRC-1 mutant vector (or empty vector). Cells were treated with leptin (200 ng/ml, 15 min) to induce phosphorylation of STAT3 and interactions between the pSTAT3 and total SRC-1 were determined by CoIP experiments using anti-pSTAT3 and anti-SRC-1 antibodies. (e) Quantification of pSTAT3 inputs (IP: pSTAT3, IB: pSTAT3, n=4-8). (f) Neuro2A cells were co-transfected with leptin receptor vector, a POMC luciferase expression reporter construct, and wild-type human SRC-1 (or empty vector). Cells were stimulated with 200ng/ml leptin or vehicle for 15 minutes and then incubated for 6 hours, following which luminescence was measured (n=5/6). (g) Neuro2A cells were co-transfected with leptin receptor vector, a POMC luciferase expression reporter construct, and wild-type human SRC-1, a dominant negative STAT3 mutant, or their combination. Cells were all stimulated with 200ng/ml leptin for 15 minutes and then incubated for 6 hours, following which luminescence was measured (n=3);* $P<0.05$ and *** $P<0.001$ in two-way ANOVA followed by pairwise tests with a Sidak adjustment. Data were presented as mean ± SEM. **Source data are provided as Supplementary Data 3.xlsx.**

Supplementary Figure 4. Interactions between mutant SRC-1 and nuclear receptors.

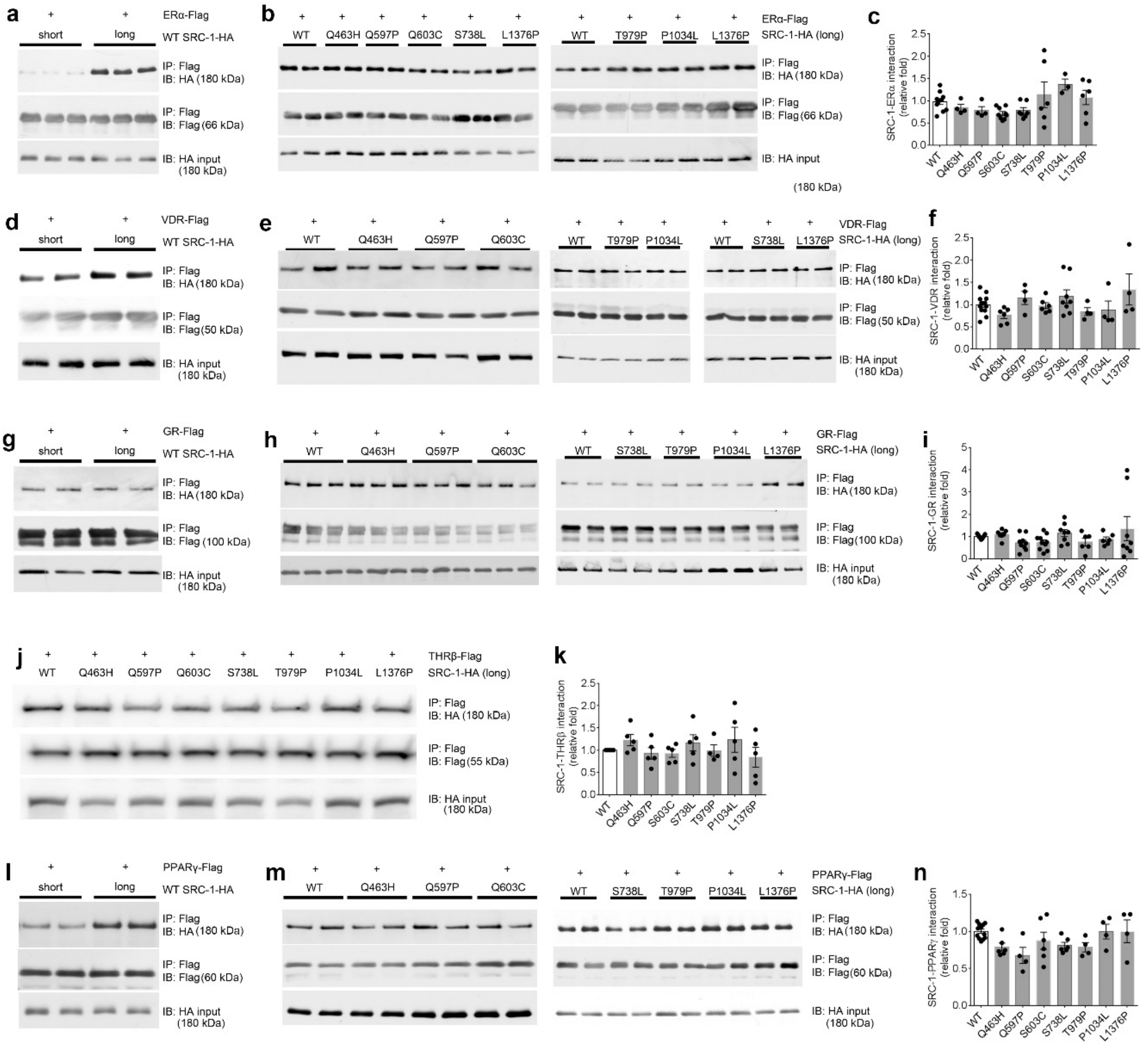

Numbers of experiments are indicated; data are presented as mean ± SEM and compared using one-way ANOVA followed by post hoc Sidak tests. (a) HEK293 cells were transfected with ERα-Flag and treated with 17β-estradiol (0.2 μg/ml, 30 min) to stimulate ERα. ERα was purified and incubated with the short or long isoform WT SRC-1-HA vectors, and interactions between the ERα and SRC-1 were determined by CoIP experiments using anti-Flag and anti-HA antibodies. (b-c) HEK293 cells were transfected with ERα-Flag and were treated with 17β-estradiol (0.2 μg/ml, 30 min) to stimulate ERα. ERα was purified and incubated with the SRC-1 (long isoform WT or one of the 7 mutants), and interactions between the ERα and SRC-1 were determined by CoIP experiments using anti-Flag and anti-HA antibodies. (b) Representative blots showing interactions between ERα and SRC-1 (WT or all 7 mutants), and inputs of ERα and SRC-1-HA. (c) Summary quantification for WT and all 7 SRC-1 mutants. Comparative folds were calculated as the ratios of HA blots and HA inputs (n=3-10). (d) HEK293 cells were transfected with VDR-Flag and treated with 1,25-dihydroxyvitamin D_3 (0.2 μM, 30 min) to stimulate VDR. VDR was purified and incubated with the short or long isoform WT SRC-1, and interactions between the VDR and SRC-1 were determined by CoIP experiments using anti-Flag and anti-HA antibodies. (e-f) HEK293 cells were transfected with VDR-Flag and treated with 1,25-dihydroxyvitamin D_3 (0.2 μM, 30 min) to stimulate VDR. VDR was purified and incubated with SRC-1 (long isoform WT or one of the 7 mutants), and

interactions between the VDR and SRC-1 were determined by CoIP experiments using anti-Flag and anti-HA antibodies. (e) Representative blots showing interactions between VDR and SRC-1 (WT or all 7 mutants), and inputs of VDR and SRC-1-HA. (f) Summary quantification for WT and all 7 SRC-1 mutants (n=4-12). (g) HEK293 cells were transfected with GR-Flag and treated with dexamethasone (10 µM, 30 min) to stimulate GR. GR was purified and incubated with the short or long isoform WT SRC-1, and interactions between the GR and SRC-1 were determined by CoIP experiments using anti-Flag and anti-HA antibodies. (h-i) HEK293 cells were transfected with GR-Flag and treated with dexamethasone (10 µM, 30 min) to stimulate GR. GR was purified and incubated with SRC-1 (long isoform WT or one of the 7 mutants) and interactions between the GR and SRC-1 were determined by CoIP experiments using anti-Flag and anti-HA antibodies. (h) Representative blots showing interactions between GR and SRC-1 (WT or all 7 mutants), and inputs of GR and SRC-1-HA. (i) Summary quantification for WT and all 7 SRC-1 mutants. Comparative folds were calculated as the ratios of HA blots and HA inputs (n=6-10). (j-k) Cell lysate from THRβ-Flag transfected HEK-293 cells were incubated with HA-SRC-1 cell lysate (long isoform WT or one of the 7 mutants) overnight with 1 µM T3 thyroid hormone. Interactions between THRβ and SRC-1 were determined by CoIP experiments using anti-Flag and anti-HA antibodies. (j) Representative blots showing interactions between THRβ and SRC-1 (WT or all 7 mutants), and inputs of THRβ and SRC-1-HA. (k) Summary quantification for WT and all 7 SRC-1 mutants. Comparative folds were calculated as the ratios of HA blots and HA inputs (n=4/5). (l) HEK293 cells were transfected with PPARγ-Flag and treated with rosiglitazone (50 µM, 30 min) to stimulate PPARγ. PPARγ was purified and incubated with the short or long isoforms of WT SRC-1, and interactions between the PPARγ and SRC-1 were determined by CoIP experiments using anti-Flag and anti-HA antibodies. (m-n) HEK293 cells were transfected with PPARγ-Flag and treated with rosiglitazone (50 µM, 30 min) to stimulate PPARγ. PPARγ was purified and incubated with the long isoform of SRC-1 (WT or one of 7 mutants), and interactions between the PPARγ and SRC-1 were determined by CoIP experiments using anti-Flag and anti-HA antibodies. (m) Representative blots showing interactions between PPARγ and SRC-1 (WT or all 7 mutants), and inputs of PPARγ and SRC-1-HA. (n) Summary quantification for WT and all 7 SRC-1 mutants (n=4-12). **Source data are provided as Supplementary Data 4.xlsx.**

Supplementary Table 1. Primer sequences.

Gene/Protein	Species	Primer Sequence	Primer Names	Accesson#
Cyclophilin	mouse	5`tggagagcaccaagacagaca	CYCLO-QF	M60456
		5`tgccggagtcgacaatgat	CYCLO-QR	
POMC	mouse	5'gaggccactgaacatctttgtc	mPOMC-QF	NM_008895
		5'gcagaggcaaacaagattgg	mPOMC-QR	
SOCS3	mouse	5'cacctggactcctatgagaaagtg	mSOCS3-QF	NM_007707
		5'gagcatcatactgatccaggaact	mSOCS3-QR	
POMC-promoter	mouse	5'gtttgggagcttggtgtgtt	POMC-F1	
Site 1		5'ggtgcctgcctaatctacca	POMC-R1	
POMC-promoter	mouse	5'ttcccatcattggggaaatc	POMC-F2	
Site 2		5'tcttgcagatcggagtggaa	POMC-R2	
POMC-promoter	mouse	5'gagacagaggcccagacatttt	POMC-F3	
Site 3		5'ccgagaatgaaagttgtggtgaa	POMC-R3	

KEY RESOURCES TABLE

REAGENT or RESOURCE	SOURCE	IDENTIFIER
**Antibodies**		
rabbit anti-pSTAT3 antibody	Cell Signaling	9145s
biotinylated anti-rabbit secondary antibody	Vector Labs	BA-1000
rabbit monoclonal SRC-1 (128E7) antibody	Cell Signaling	2191s
monoclonal anti-β-Actin antibody (AC-15)	Sigma	A5441
Peroxidase-AffiniPure Goat Anti-Mouse IgG (H+L)	Jackson ImmunoResearch	115-035-166
Peroxidase-AffiniPure Goat Anti-Rabbit IgG (H+L)	Jackson ImmunoResearch	111-035-144
Stat3 Antibody (C-20)STAT3	Santa Cruz	sc-482
Monoclonal ANTI-FLAG® M2 antibody	Sigma	F3165
**Chemicals, Peptides, and Recombinant Proteins**		
tamoxifen	sigma	T-5648
leptin	HARBOR-UCLA Research And Education Institute	N/A
17β-estradiol	Sigma	
Vitamin D3 (Calcitriol)	TOCRIS	2551
dexamethasone	Sigma	D4902
rosiglitazone	ADIPOGEN	AG-CR1-3570
**Experimental Models: Cell Lines**		
SRC-1 KO MEF	This paper	N/A
**Experimental Models: Organisms/Strains**		
Mouse: Pomc-Cre	Jackson Laboratory	005965
Mouse: Pomc-CreER	Berglund, et al. 2013	N/A
Mouse: SRC-1$^{lox/lox}$	Yamada et al., 2004	N/A
Mouse: Rosa26-tdTOMATO	Jackson Laboratory	007909
Mouse: NPY-GFP	Jackson Laboratory	006417
Mouse: SRC-1$^{L1376P/+}$	This paper	N/A
Mouse: SRC-1-KO	Xu et al., 1998	N/A
**Recombinant DNA**		
Psg5 SRC-1	Louet, et al., 2010	N/A
pRc/CMV-STAT3; STAT3C-Flag	Horvath, et al., 1995	N/A
pcDNA3.1-LepR	This paper	N/A
pGL3-SOCS3-Luc 6T1	Auernhammer et al., 1999	N/A
pGL3-rPOMC-Luc	Zhang, et al., 2011	N/A
pGL3-hPomc-Luc	This paper	N/A
pcDNA3.1-hSRC-1-HA	This paper	N/A
pcDNA3.1-hSRC-1-Myc	This paper	N/A
pcDNA3.1-hERα-Flag	This paper	N/A
pcDNA3.1-hPPARγ-Flag	This paper	N/A
pcDNA3.1-hGR-Flag	This paper	N/A
pcDNA3.1-hGR-Flag	This paper	N/A
pRL-SV40	Promega	E2231
