# Supplementary material for: Steroid receptor coactivator-1 modulates the function of Pomc neurons and energy homeostasis
Source: Nat Commun. 2019 Apr 12;10:1718. doi: 10.1038/s41467-019-08737-6 (PMC6461669; doi:10.1038/s41467-019-08737-6)
Supplement: Supplementary file 3 — Description of Additional Supplementary Files [file 41467_2019_8737_MOESM3_ESM.pdf]

Description of Additional Supplementary Files

File Name: Supplementary Data 1
Description: Source Data file for Supplementary Figure 1

File Name: Supplementary Data 2
Description: Source Data file for Supplementary Figure 2

File Name: Supplementary Data 3
Description: Source Data file for Supplementary Figure 3

File Name: Supplementary Data 4
Description: Source Data file for Supplementary Figure 4
