# Supplementary figures and images for: Steroid receptor coactivator-1 modulates the function of Pomc neurons and energy homeostasis
Source: Nat Commun. 2019 Apr 12;10:1718. doi: 10.1038/s41467-019-08737-6 (PMC6461669; doi:10.1038/s41467-019-08737-6)

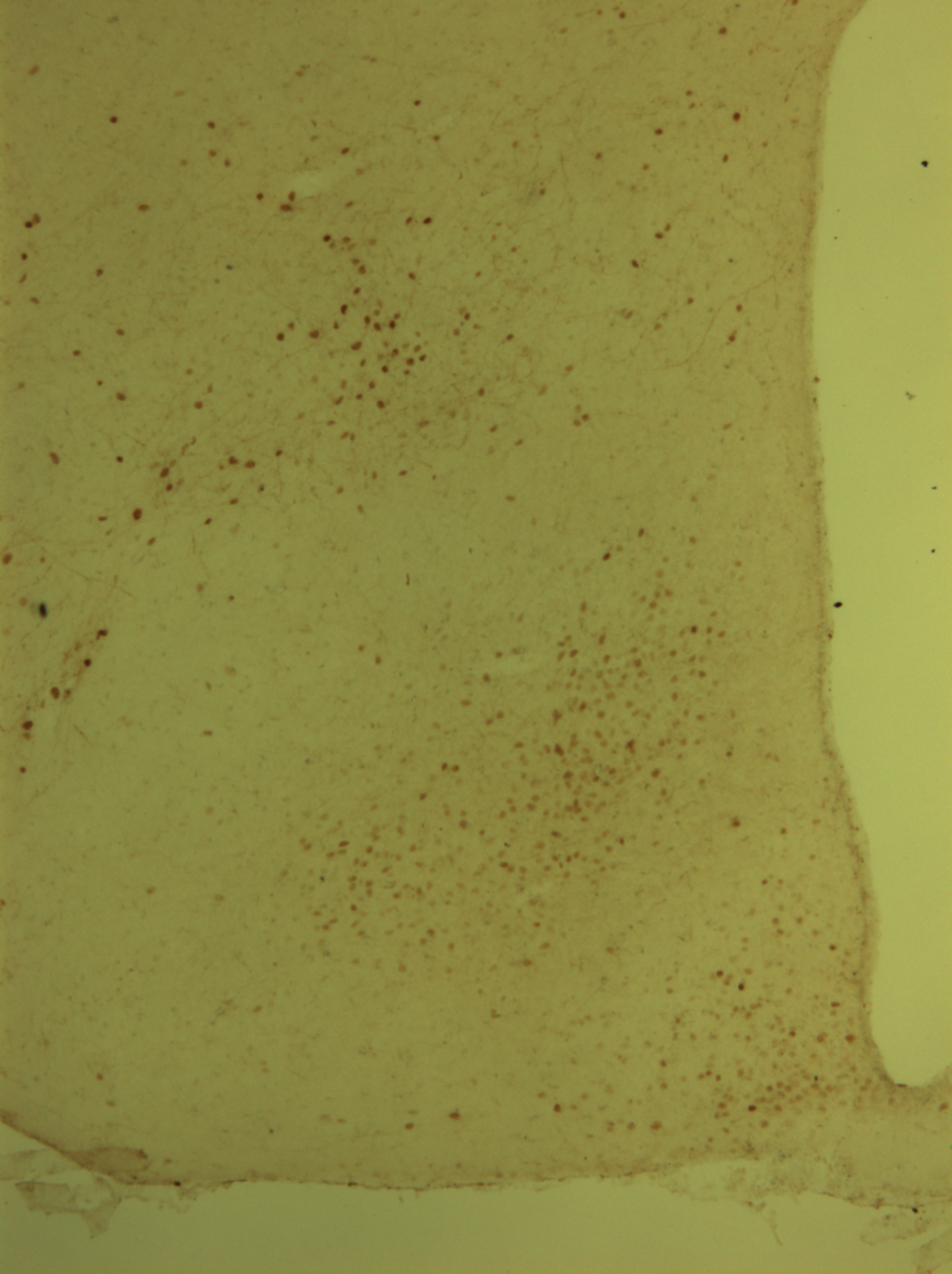

Supplement: Supplementary file 11 — Source Data Figure 2E [file 41467_2019_8737_MOESM11_ESM.tif]

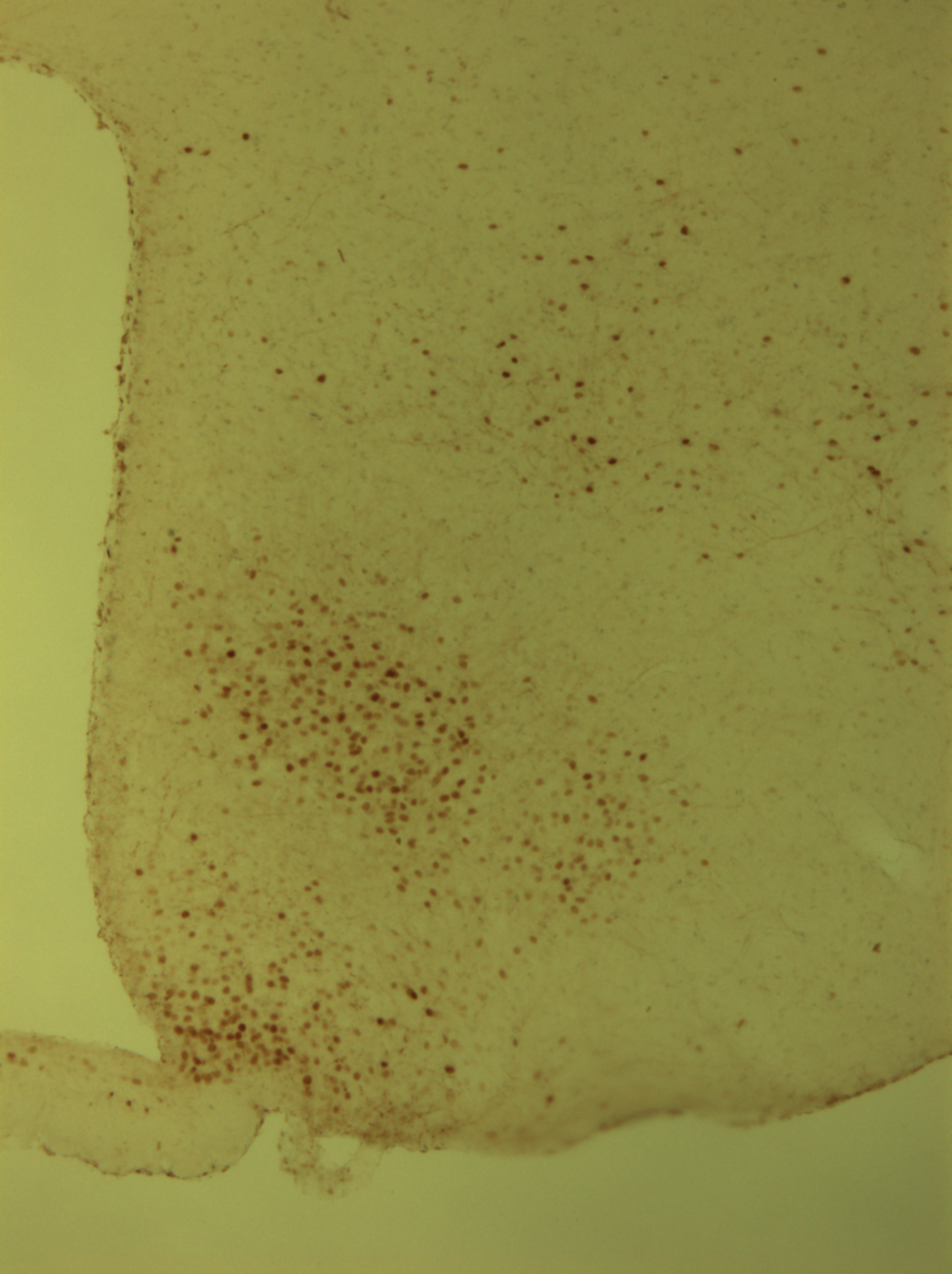

Supplement: Supplementary file 12 — Additional Source Data Figure 2E [file 41467_2019_8737_MOESM12_ESM.tif]
